# Draft Genome Sequences of Two Strains of *Paenibacillus glucanolyticus* with the Ability To Degrade Lignocellulose

**DOI:** 10.1128/genomeA.00423-16

**Published:** 2016-06-23

**Authors:** Stephanie L. Mathews, Joel Pawlak, Amy M. Grunden

**Affiliations:** a Department of Biology, Campbell University, Buies Creek, North Carolina, USA; b Department of Forest Biomaterials, North Carolina State University, Raleigh, North Carolina, USA; c Department of Plant and Microbial Biology, North Carolina State University, Raleigh, North Carolina, USA

## Abstract

*Paenibacillus glucanolyticus* 5162, a bacterium isolated from soil, and *Paenibacillus glucanolyticus* SLM1, a bacterium isolated from pulp mill waste, can utilize cellulose, hemicellulose and lignin as sole carbon sources for growth. These two strains of *Paenibacillus glucanolyticus* were sequenced using PacBio and Illumina MiSeq technologies.

## GENOME ANNOUNCEMENT

*Bacillus glucanolyticus* was first isolated from environmental soil samples by Alexander and Priest (1). This Gram-positive, facultative anaerobic bacterium is characterized by its terminal spore formation, motile colonies, and ability to degrade a variety of β-glucans (1). *B. glucanolyticus* can hydrolyze carboxymethyl cellulose, curdlan, pustulan, and xylose (1, 2) and was renamed *Paenibacillus glucanolyticus* in 1997 by Shida et al. (3) based on 16S rRNA gene similarity. A microorganism that was recently isolated from lignin-containing pulping waste black liquor was identified to be *P. glucanolyticus* (designated SLM1). This strain was determined to have optimal growth at 37°C and pH 9.0 (4), to hydrolyze cellulose and hemicellulose, and to grow on lignin as the sole carbon source under aerobic and anaerobic conditions (5). The ability to degrade lignin suggests that *P. glucanolyticus* contains genes which encode enzymes that are involved in lignin depolymerization, and their elucidation will provide additional information about enzymes involved in bacterial lignin degradation under basic and anaerobic conditions.

*P. glucanolyticus* SLM1 and type strain 5162 genomic DNA were submitted to the Duke University Sequencing and Genomic Technologies Resource for PacBio single-molecule real-time (SMRT) sequencing (RSII platform). Each DNA library was prepared using two SMRT cells, resulting in 300,584 raw reads with a mean read length of 5,753 bases, totaling 1,729,341,910 bases for strain SLM1. Type strain 5162 had 300,584 raw reads with a mean read length of 5,120 bases, totaling 1,539,234,297 bases. Generated reads were introduced into the Hierarchical Genome Assembly Process (HGAP), assembled with the Celera Assembler, and polished with Quiver. Resulting assemblies produced three contigs for strain SLM1 with a total genome size of 7,039,886 bp, while assembly of 5162 produced one contig with a total genome size of 6,217,882 bp. Library preparation and sequencing were also performed by the Genomic Sciences Lab at North Carolina State University. Each strain was sequenced using Illumina MiSeq v3 chemistry. The two strains were combined in one MiSeq library and generated 26,000,000 reads for each strain, with an average sequence length of 285 bases. Reads produced by Illumina MiSeq were assembled into large PacBio contigs using SeqMan NGen (DNAStar.v.11.0). The 7-Mb genome has a total GC content of 44.99% with 99 RNAs (rRNAs and tRNAs) (6). The 6.2-Mb genome has a total GC content of 43.50% with 98 RNAs (rRNAs and tRNAs). Annotation was performed using Rapid Annotation using Subsystem Technology (RAST) (7). RAST predicted 6,480 coding sequences for SLM1 and 5,675 coding sequences for 5162.

Draft genomes of *P. glucanolyticus* SLM1 and 5162 will assist in determining metabolic pathways by which these bacteria break down lignocellulosic components and other compounds that may be of biological/biotechnological importance. Physiological differences have been observed between the two strains with respect to their ability to degrade lignocellulose. Genomic sequence comparison of the two genomes found 89.47% similarity between these strains.

### Nucleotide sequence accession numbers.

These whole-genome assemblies have been deposited at DDBJ/EMBL/GenBank under accession numbers LWMH00000000 and CP015286. The versions described in this paper are the first versions.
